# University Clinique

**Published:** 1854-04

**Authors:** S. D. Gross

**Affiliations:** Professor of Surgery in the University of Louisville


### Art. II.—UNIVERSITY CLINIQUE.—CASES AND OBSERVATIONS IN SURGERY.

BY S. D. GROSS, M. D., PROFESSOR OF SURGERY IN THE UNIVERSITY OF LOUISVILLE.

### Case 4.—*Urinary Calculus; Incontinence of Urine for Sixteen Months; Lithotomy; Cure.*

The patient whom I now present to you is laboring under calculus of the bladder, attended with certain symptoms which invest the case with peculiar interest. His name is Harvey Williams. He is fifty-three years of age, a farmer by occupation, married and the father of ten children, being a resident of Newberg, Warrick county, Indiana. About two years ago he began to experience some pain in urinating, more especially during the latter stages of the process, accompanied with a burning sensation at the neck of the bladder and along the course of the urethra. The symptoms lasted for about five months, when he was seized with complete incontinence of urine, which has lasted uninterruptedly until the present moment, a space of sixteen months. Owing to his infirmity, the man has been constantly wet and uncomfortable, and has never been able to lie down at night. His habit is to sleep in an arm-chair, with a vessel under him to receive the urine as it flows from the bladder. You will notice that he wears a short apron, the object of which is, as he informs me, to cover the penis, which, in consequence of the incessant dribbling of urine, he carries constantly on the outside of his pantaloons.

The pain in urinating, the symptom which first attracted his attention, has been gradually increasing in violence, and is frequently paroxysmal in its character; it is generally attended with griping and tenesmus, and darts about in different directions through the pelvic region, the perinæum, groins, and thighs. The recumbent posture usually affords him some relief, but the moment he gets up, sits down, or walks about, his sufferings return with their accustomed severity. Owing to the frequent straining which attends the urinary distress, there has been, for some time past, prolapsus of the bowel, which the patient says he frequently returns with his finger. He has passed a great many little calculi, varying in size from that of a hemp-seed to that of a small pea, the discharge being always attended with an increase of the local suffering, proportioned to the volume of the concretion and the sensibility of the urethra. During the early stage of his complaint, before he lost his control over the bladder, the calls to urinate were frequent and very urgent, but since the fluid has been dripping off his suffering has been less, except when his water is involuntarily forced away, as it often is, in a strong stream. Formerly the flow of urine was often interrupted as if something had suddenly blocked up the passage. The man discharges a great deal of ropy mucus and occasionally pieces of clotted blood.

Considering the amount and character of the local suffering, it is surprising what little derangement there is in the general health. Still the disease has left its impress upon the system, and that in a marked degree. The countenance is pale and care-worn, and there is, at times, a great deal of mental depression. The pulse, however, is soft and of natural frequency, the appetite is good, and there is seldom any febrile movement. The skin and bowels act sufficiently well. The sleep is, of course, interrupted, but he has often snatches of several hours' duration, and in the day and night he generally obtains as much as men commonly do in health.

My object in bringing this man before you this morning is to show you his appearance, to describe his symptoms, and to sound him, with a view of ascertaining the real condition of his urinary organs. He has all the rational signs of urinary calculus, but to determine whether he is really laboring under this disease, it is absolutely necessary to explore his bladder; for no surgeon, who has any self-respect, or who has the slightest regard for the welfare of his patient, would think of performing so serious an operation as lithotomy without such a precaution. You should never do anything without a proper reason. To cut an individual for stone, simply because he has some of the symptoms of stone, when you are not satified of the fact from a thorough exploration of the bladder, would be the height of folly, and would justly subject you to the ridicule and contempt of your professional brethren. Unfortunately such mistakes have occasionally been committed even after the use of the sound, and that, too, by men who, from their position, ought to have known better. I am acquainted with the particulars of two cases in this city, in which lithotomy was performed by the same operator, without a calculus having been found. I would not be guilty of such an error for the whole State of Kentucky. Last summer a friend in New York wrote to me that he had just returned from London, where he had spent some time in visiting the different medical institutions. At one of the great hospitals, whither he had been attracted by the fame of its surgeons, he witnessed, one day, the operation of lithotomy; but, to his surprise, the patient, after having been upon the table for a whole hour, was untied, and carried to his bed without any stone having been found. It is hardly necessary to add that my friend went away with no very favorable impression of the operator's ability as a diagnostician.

In sounding this patient I shall place him upon his back, his head resting upon a pillow, and his lower extremities, divested of clothing, being slightly separated from each other and raised towards the pelvis. A steel instrument, termed a sound, fashioned like a catheter, and furnished with a perfectly smooth and rather large handle, is then introduced into the bladder and moved about in different directions, so as to bring its point and curved part, if possible, in contact with the foreign body. The object is not merely to feel the stone, but, by percussing it, to elicit the peculiar metallic sound, technically denominated a *click*, so characteristic of the presence of a urniary concretion. When these two signs exist, no surgeon can, for a moment, entertain any doubt respecting the real nature of the case; they are positive, unequivocal, pathognomonic. Both these signs exist in the case befere us. The moment the instrument enters the bladder it touches the stone and produces the peculiar noise adverted to. The concretion is apparently of large size, but of this we cannot be positively certain, because the instrument cannot, in consequence of the contracted and sensitive condition of the bladder, be inserted to any distance, so as to enable us to measure its volume.

In sounding there should always be a certain quantity of water in the bladder; not too little, on the one hand, lest the bladder should, by its contraction, impede the movements of the instrument, and not too much, on the other, lest the concretion be lost in the redundant fluid. On an average the quantity should not exceed five or six ounces. The proper procedure is to request the patient to hold his urine several hours before the contemplated examination, or, when this is impracticable, to inject the requisite quantity by means of the catheter and syringe. In the present case, unfortunately, the patient can neither hold his urine, nor can he, in consequence of the size and situation of the calculus, and the contracted and irritable state of the bladder, tolerate the latter operation. This, however, is to be the less regretted, because the diagnosis could not possibly be more clear and satisfactiory if we could see the stone in the bladder, or hold it in our hand.

Sounding should never be performed the moment the patient comes under our charge, especially if he has undergone a fatiguing journey. On the contrary, he should always be subjected to a sort of preliminary treatment, consisting of rest in the horizontal posture, light diet, and purgatives, with, perhaps, the warm bath, and, in most cases, the use of soda and infusion of uva ursi and hops. The latter means are particularly indicated when there is unusual irritability of the bladder, with great pain and distress in voiding the urine. It is wonderful what prompt improvement this prescription commonly affords in a few days, in apparently the most unpromising cases of this description. In my hands it has often acted like a charm.

The treatment here mentioned has been pursued in the case of Williams for some days past, and we shall rigidly persevere in it for the present, or until there is reason to believe that he is in a condition to bear the operation necessary for his relief.

It was doubtful for several weeks whether Williams really was a fair subject for lithotomy, such was the state of his bladder and of his general health. Determined, however, to afford him the only chance that presented itself for his relief, I continued to give him daily attention, until, finding that his condition was much improved, I requested the opinion of my collegues upon the case, and as many as attended the consultation, ROGERS, PALMER, and FLINT, all concurred in the propriety of a resort to the knife. The pulse at this time was perfectly natural in point of volume and frequency, the appetite and sleep were good, and the bladder was much more patient of its contents. This was on a Thursday. The operation was now appointed for the following Saturday, but early on that day, in consequence of some misgivings about its result, the man disappeared from the Infirmary, and nothing more was heard of him for nearly three weeks, except incidentally, that he had gone home, a distance of one hundred and twenty miles, having travelled the greater part of the road on foot during very cold weather. Notwithstanding the fatigue and suffering endured during this journey, which it took him eight days to complete, the man, upon his return to Louisville, was decidedly improved in health, and in every respect in as good a condition for an operation as at the time of his unceremoneous departure.

The patient being fully determined to submit to the operation, I shall now proceed to perform it, premising a few remarks upon the different steps of it.

It is not necessary to say anything here respecting the manner of securing the patient. The object of the procedure is to render him as passive as possible, and to enable the assistants, who have charge of the limbs, to expose the perinæum, as it lies upon the edge of the table, to the greatest advantage to the operator. Chloroform is next administered, and as soon as its full effects are apparent, a staff—a steel instrument, shaped like a catheter, and having a very wide and deep groove, with a long, broad and rough handle—is introduced into the bladder, and confided to an assistant standing on the left side of the patient. This assistant also takes charge of the scrotum, holding it out of the way of the operator. The staff should be as large as the urethra will readily admit; the handle should be inclined a very little towards the right side of the median line, and be held in such a manner as to form almost a right angle with the body. The curved part of the instrument should be hooked up against the arch of the pubes, otherwise there will be danger of wounding the rectum. Most lithotomists recommend the staff to be introduced before tying the patient's feet; but such a practice is, on many accounts, objectionable, and I never adopt it. The stone having been touched with the staff, to assure ourselves of its presence, the perinæum shaved, and every assistant instructed in regard to his duties, the operator, sitting upon a low chair, or resting himself, as I generally do, upon one knee, stretches the integuments of the upper part of the perinæum, and enters his knife, a long and slender scalpel, about fifteen lines above the anus, beginning at the raphé and terminating a short distance below the margin of the anus, nearly midway between this outlet and the tuberosity of the ischium, but a little nearer the latter than the former. The incision in the adult is at least three inches in length, and it embraces the skin, the cellulo-adipous substance, and the superficial fascia; its direction being obliquely downwards and outwards. Re-inserting the knife into the upper angle of the wound, the operator next divides the transverse perinæal muscle and artery, the deep-perinæal ligament, and the membranous portion of the urethra, the opening into the latter being large enough to admit the point of the fore-finger of the left hand. In the third and last stage of the proceeding, the operator divides the remainder of the membranous portion of the urethra, the left lobe of the prostate gland and the neck of the bladder, in a direction obliquely downwards and outwards; care being taken to lateralize the knife properly, and to push the rectum over towards the right side with the finger as it lies in the upper part of the wound, lest this tube should be accidentally injured. The moment the bladder is incised the urine gushes out, thus apprising the surgeon of his progress, and at the same time forcing down the calculus to the neck of the organ. The staff is now withdrawn and the finger pushed on into the wound, which, if necessary, may now be dilated by the pressure of the finger, or, if deemed prudent, with a probe-pointed bistoury. In general I prefer the former, as less likely to produce hemorrhage and other mischief. Keeping the finger in the bottom of the wound, the forceps are passed over its surface into the bladder, where they are used first as a searcher, and next for grasping the calculus. Having accomplished this object, the extraction is effected very much as the head of the child in parturition; that is, in the direction of the artificial outlet, and by moving the instrument *gently* and *coaxingly* from side to side, lest undue violence be inflicted upon the parts, especially the neck of the bladder and the prostate gland. It sometimes happens that the calculus is seized in its large diameter; when this is the case, the hold must be relinquished, and the direction of the concretion changed. For want of this precaution great difficulty is sometimes experienced in this stage of the operation, and great, if not irreparable, injury inflicted upon the parts involved in the operation. If several calculi are present they are seized and extracted one after another. Fragments and small concretions are generally best removed with the scoop. Clearance having thus been effected, the bladder is freely syringed with tepid water, thrown in with some degree of force, that it may reach every part of the organ, and thus effectually wash away every particle of calcareous matter. This being done, and seeing that there is no hemorrhage, the patient is liberated from his constrained position and put in bed.

In performing the lateral operation there are several circumstances which claim particular attention, because they exert a most important influence, either for good or evil, upon the result of the case. The wound, considered in its ensemble, will be found to resemble a truncated cone, the base of which corresponds with the surface of the perinæum, while the apex is represented by the neck of the bladder. Now the rule is, in all cases, to make the base large and the apex small; or, in other words, to have a free and dependent external opening, and a narrow internal one. By this procedure a ready outlet is afforded to the urine, at the same time that this fluid is prevented from insinuating itself round the prostate gland and neck of the bladder; an occurrence which is always followed by disastrous effects, either eventuating in sloughing of the parts or in the death of the patient. The division of the prostate gland, in its entire length, can be necessary only when the foreign body is of extraordinary dimensions, and even then it is questionable whether it would not be better to break the concretion than to subject the parts to the risk of infiltration.

Another circumstance, already incidentally mentioned, is not to use the knife too freely in dividing the deep-seated structures, but instead of this to employ the finger, with which they may often be easily torn to any extent that may be desired. This is particularly true in regard to young children, in whom these structures are always more or less dilatable and lacerable. The object of this procedure is to prevent urinary infiltration and undue injury to the vessels; two circumstances so liable to happen when the parts are freely divided with the knife.

I do not deem it necessary or even proper to retain a tube in the bladder, to conduct off the urine during the first few days after the opetation. The practice, which dates as far back as the time of Colot, in the early part of the last century, has been warmly advocated by several modern lithotomists, especially the late Mr. Liston, but without, as I conceive, any just reason. Indeed, so far from doing good, I think it may generally be regarded as tending to do harm, by exciting undue irritation both in the wound and the mucous membrane of the bladder, attended with pain and spasm in the neck of this organ. It is for this reason that I never employ it; and, as to the infiltraiion of urine which it is designed to prevent, I am happy to say that I have never met with it in my own practice after any operation of this kind. Nor is it necessary to tie the patient's legs together after he has been placed in bed; on the contrary, I always permit him to adopt whatever posture he may choose or find most agreeable.

There is sometimes considerable hemorrhage after this operation, and the blood, as you may suppose, may proceed from various sources. In the case of Williams there was but little bleeding at the time of the operation; about three hours afterwards, however, I was requested to visit him, and found a clot of blood, about the size of a small fist, between his thighs upon the draw-sheet. Upon examining the parts, with the aid of several of my young friends, the blood was seen issuing slowly from the bottom of the wound, evidently from the prostate gland and neck of the bladder, the vessels of which, especially the veins, had, doubtless, become very much enlarged under the irritation occasioned by the constant lodgment here of the calculus. Finding that no ligatures could be employed, I contented myself with introducing into the wound, as far back as possible, a piece of soft sponge, dipped in powdered alum, and in confining this firmly by means of a large compress and a T bandage. No hemorrhage showed itself after this treatment. The urine soon began te pass off by the side of the tampon, which was expelled spontaneously about sixty hours after its introduction, and, excepting some degree of abdominal tenderness, on the second day after the operation, the case went on without any untoward symptoms. The recovery, however, has been very tardy, and even now, at the end of several weeks, a considerable portion of the urine still passes through the wound, though this is, apparently, almost healed. These circumstances I ascribe to the fact that, during the first month after the operation, there was hardly any attempt at re-union, the wound being large and gaping, and the granulations pale and flabby, and all this despite the care which was taken to improve the general health by the employment of milk-punch, nutritious food and other means. About the end of this time, when the parts seemed to be doing well, there was, one day, a sudden discharge of thick, greenish-looking matter from the wound, so copious and offensive as to convince me that it must have proceeded from an abscess about the prostate gland and neck of the bladder. The flow soon ceased, and the patient did not appear to have experienced any ill effects from it in any way. During the last few weeks his improvement has been quite satisfactory, and there is reason to believe that the recovery, although slow, will be complete. I am induced, however, to believe, from the fact that there is occasionally a slight discharge of pus, that there may be ulceration of the mucous membrane of the bladder, or organic disease of the kidneys, and, should this conjecture be true, then all our hopes in this respect will be very likely to be disappointed.* The urine, before the operation, was generally alkaline and surcharged with thick, ropy mucus. Since the operation the mucus has greatly diminished in quantity, and instead of being alkaline it has, at times, been remarkably acid. The calculus, according to Prof. SILLIMAN, consists, externally, of phosphate of magnesia.

---

CASE 5.—*Calculus in the Bladder; Lithotomy; Cure.*

A week ago I performed in your presence the lateral operation of lithotomy upon a man named Williams, from the State of Indiana. I am happy to inform you that he is doing well, and that everything about the case augurs a favorable termination. To-day it is my intention to perform a similar operation upon a man from the vicinty of Madisonville, Hopkins county, Kentucky. His age is forty-three; he is married, and is the father of ten children; his habits are strictly temperate; his occupation is farming, and his name is David A. Leech. He resides in a coal region, and the water which he uses in his family is partly lime and partly free-stone. About thirteen years ago he was attacked with a severe pain in the region of the kidneys, extending down into the testicles, and accompanied by extreme nausea and vomiting. He states that he had several paroxysms of this kind in pretty rapid succession, and that he was invariably relieved by a dose of purgative medicine. A short time after these attacks he experienced some uneasiness in the neck of the bladder, which continued to within three years of the present time. It then became much more aggravated, and was attended with frequent and urgent calls to void his urine, which passed off involuntarily. The pain extends along the entire track of the urethra, and is much increased

* Since the above was written, this patient has entirely recovered.

by walking and riding on horse-back. There is a constant soreness at the neck of the bladder, and he has occasionally discharged small portions of blood and also little calculi. The general health is little, if any, impaired.

Upon sounding this patient in the latter part of October, the instrument immediately came in contact with a calculus, which, from the sensation communicated to my finger, I supposed to be considerably under the medium size. In the operation a small fragment was broken off and discharged soon afterwards. Presuming it might be practicable to crush the calculus, I requested the patient to remain perfectly quiet, to live as lightly as possible, to take an occasional purge, and to drink, four times a day, at equal intervals, a small wine glassful of uva ursi and hop tea, with about thirty grains of bicarbonate of soda in each dose. A large catheter was inserted into the bladder every day, in order to render that organ tolerant of the presence of instruments and to dilate the urethra. Some time having been spent in this manner, I introduced Jacobson's lithotriptor, but, owing to the restlessness of the patient, growing out of his excessive irritabilty, I was obliged to desist before I could succeed in grasping the foreign body. About a week afterwards the operation was repeated with no better success. The last attempt was followed by a smart attack of cystitis, which prohibited, for the time, all further interference. Under the usual treatment the disease has gradually subsided, and the parts have sufficiently recovered to justify a resort to the knife. The operation which I shall adopt is the same as that which I performed upon Williams at our last clinique. With the nature of this operation, and the manner of executing it, the parts which are concerned in it, and the position of the patient and of the assistants, you are already familiar, and I need not, therefore, again describe them. We shall give the patient chloroform, as in the previous case, and when he is thoroughly influenced we shall proceed to our task.

The calculus, as you have no doubt noticed, was seized and extracted with great facility, its volume, which is small, having been conducive to this end. The bladder having been washed out with tepid water, thrown in with a large syringe, the patient was kept for a short time upon the table to see whether there would be any hemorrhage. Finding that more blood issued from the wound than was proper, the edges were separated with a view of discovering its source. The greatest amount seemingly proceeded from the deep portion of the wound, as was proved by the fact that, when pressure was made, especially towards the pubes, the flow was either greatly diminished or entirely arrested. The transverse perinæal artery, althougn it did not bleed much, was ligated, as was also a branch of the posterior hemorrhoidal. Finally, a ligature was applied to the artery of the bulb, when, a soft sponge being introduced into the wound, to render "assurance doubly sure," the patient was untied and put to bed.

No secondary hemorrhage ensuing, the sponge was withdrawn at the end of the second day, and the man recovered without the slightest untoward symptoms of any kind. The only medicine which he took was an occasional laxative, and now and then a small quantity of morphia, to quiet nervous irritation. The wound, as in the case of Williams, was a little longer in healing than usual, and the urine did not begin to resume its natural channel until towards the close of the fourth week. This was, doubtless, owing, mainly, to the irritation which it had experienced from the contact of the sponge, and perhaps also to the fact that I was obliged to enlarge it somewhat in my attempt to ligate the vessels above alluded to. The urine now, at the end of six weeks and a-half, is voided entirely by the natural route, the wound in the perinæum being nearly cicatrized, and the general health excellent. The urine, tested by Prof. SILLIMAN, is slightly alkaline, and contains a few crystals of soda. The man is able to retain it very well, and does not void it oftener than once every three or four hours. I have given the patient permission to go home next Monday. To guard against relapse, I shall request him to live upon plain, simple food, to regulate his bowels and secretions, to wear flannel next the skin, to avoid exposure to wet and cold, and to take, twice a day, about two hours after dinner and supper, in a third of a tumblerful of cistern water, thirty grains of bicarbonate of soda, to prevent the formation of flatulence and acidity in the alimentary canal.

